# Guidelines for the Gamification of Self-Management of Chronic Illnesses: Multimethod Study

**DOI:** 10.2196/games.7472

**Published:** 2017-05-12

**Authors:** Alaa AlMarshedi, Gary Wills, Ashok Ranchhod

**Affiliations:** ^1^ University of Southampton School of Electronics and Computer Science Southampton United Kingdom; ^2^ University of Southampton Winchester School of Art Winchester United Kingdom

**Keywords:** gamification, health care, behavior change, self-management, chronic illnesses

## Abstract

**Background:**

Gamification is the use of game elements and techniques in nongaming contexts. The use of gamification in health care is receiving a great deal of attention in both academic research and the industry. However, it can be noticed that many gamification apps in health care do not follow any standardized guidelines.

**Objective:**

This research aims to (1) present a set of guidelines based on the validated framework the Wheel of Sukr and (2) assess the guidelines through expert interviews and focus group sessions with developers.

**Methods:**

Expert interviews (N=6) were conducted to assess the content of the guidelines and that they reflect the Wheel of Sukr. In addition, the guidelines were assessed by developers (N=15) in 5 focus group sessions, where each group had an average of 3 developers.

**Results:**

The guidelines received support from the experts. By the end of the sixth interview, it was determined that a saturation point was reached. Experts agreed that the guidelines accurately reflect the framework the Wheel of Sukr and that developers can potentially use them to create gamified self-management apps for chronic illnesses. Moreover, the guidelines were welcomed by developers who participated in the focus group sessions. They found the guidelines to be clear, useful, and implementable. Also, they were able to suggest many ways of gamifying a nongamified self-management app when they were presented with one.

**Conclusions:**

The findings suggest that the guidelines introduced in this research are clear, useful, and ready to be implemented for the creation of self-management apps that use the notion of gamification as described in the Wheel of Sukr framework. The guidelines are now ready to be practically tested. Further practical studies of the effectiveness of each element in the guidelines are to be carried out.

## Introduction

The use of gamification in health care is receiving a great deal of attention in both academic research and the industry [1-13]. Gamification is the use of game elements and techniques in nongaming contexts [14]. It is employed in different areas including health care to facilitate engagement and behavioral change and increase motivation [15,16]. The attention given to gamification is due to its perceived usefulness and benefits, especially when dealing with chronic illnesses and daily self-management by patients [3,17,18]. Chronic illnesses require repetitive but important tasks that could be made easier to handle with gamification. Thus, it could be of interest for developers to gamify health and fitness apps. However, it can be noticed that many gamification apps in health care do not follow any standardized guidelines [1], which might affect the overall experience of the users. For instance, users could get bored with using a certain gamified app if it only includes gamification in an arbitrary way [19,20]. In health care apps, especially ones that target self-management of chronic illnesses, developers are advised to take into consideration many aspects of the concept of gamification and its relation to self-management.

**Figure 1:**
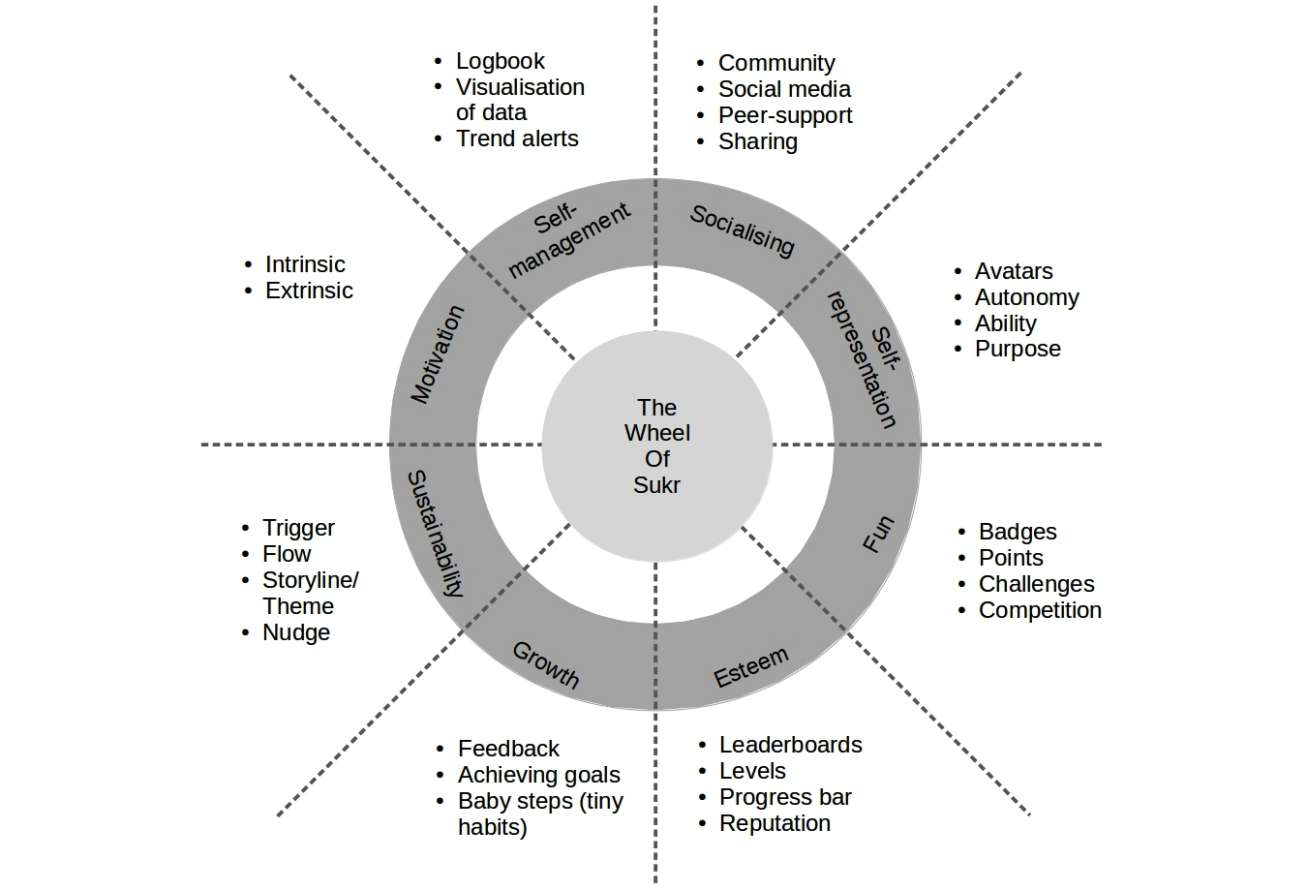
The Wheel of Sukr [21].

To overcome a shortage in the literature, we introduced the Wheel of Sukr framework [21], which is a framework for the gamification of self-management of chronic illnesses that combines behavior changing methods, game techniques, and techniques of self-management of chronic illnesses. The Wheel of Sukr consists of 8 themes: fun, esteem, growth, motivation, sustainability, socializing, self-representation, and self-management. Each theme includes a number of elements as shown in Figure 1.

The framework was validated by the authors using a mixed method study [22]. Medical doctors, psychologists, and gamification experts were interviewed and diabetic patients took part in answering a questionnaire that measures their attitudes toward the concepts covered by the framework. The results suggested that patients are keen to see self-management apps containing the concepts of the Wheel of Sukr. Also, the findings of the interviews suggest that experts see the need of gamification as represented in the framework in the area of self-management of diabetes and other chronic illnesses.

Nevertheless, the Wheel of Sukr is a theoretical framework and so is considered a high-level construct. To establish a transition from the theoretical side to the practical side, the framework and its content should be translated into a set of guidelines that can be applied practically by developers. Such guidelines should contain definitions, instructions, and suggestions that target developers who can then gamify self-management of chronic illnesses apps or systems.

This paper presents a set of guidelines based on the Wheel of Sukr framework [21,22]. The guidelines (see Multimedia Appendix 1) were assessed during expert interviews and discussed with developers in focus groups. The purpose of the expert interviews was to ensure that the guidelines represent the framework accurately and comprehensively. The purpose of the focus group discussions with developers was twofold. The first aim was to collect their views on the clarity, usefulness, and ease of implementation of the Wheel of Sukr guidelines. The second was to test if they could think of practical ways to gamify apps based on the guidelines.

## Methods

### Criteria for Creating the Guidelines

As mentioned earlier, the guidelines were built based on the Wheel of Sukr. Similar to the framework, the guidelines contain 8 themes, each of which is divided into 5 sections as shown in Table 1.

The Wheel of Sukr guidelines are designed such that they can be tailored to the goals and objectives of each app or system and its audience. For example, the type of badges and points could be different if the app is targeting children with diabetes as opposed to adults.

Experts from academia assessed the guidelines, and these assessments were discussed with developers in focus groups. The group of experts and the group of developers did not overlap in our study.

### Expert Interviews

Qualitative data were collected through expert interviews. The aim of the interviews was to conduct a formative evaluation of the guidelines to ensure that they represent the framework accurately and comprehensively. The experts were selected from the University of Southampton. They were identified and contacted in person or through email by the first author. The experts were from one or more of the following areas: game development, user experience, and gamification. The interviews were conducted by the first author.

In each interview, the interviewer started by explaining the background of the study and the framework. Next, the expert was presented with the guidelines and was asked to read one theme at a time. Next, the interviewer asked the expert to provide their evaluation and their answers to a number of open-ended questions.

Semistructured interviews were conducted with experts in developing apps and games, experts in design and user experience, and game experts in academia. The interviewer stopped conducting interviews after reaching a point of saturation. This is when data becomes redundant and no new data are found [23]. Saturation was achieved after interviewing 6 experts. The duration of each interview was 50 minutes on average, and each interview was recorded for analysis. After that, the interviews were transcribed verbatim and were analyzed using a thematic approach. The data were coded with tags that represent the 8 themes of the guidelines. Similar sets of data were identified and categorized.

**Table 1:** Sections of the guidelines.

Section	Purpose
Theme	A general construct containing elements that share the same goal.
Definition	The general idea of the theme is defined.
Goal	The purpose of the theme and its elements is stated.
Description	The theme and its elements are elaborated.
Application	The theme is translated into pointers to help in implementation.

### Focus Group Interviews

The focus group method was chosen to gather the views of developers on guideline clarity, usefulness, and ease of implementation. In particular, we wanted to find out the clarity of the content of the guidelines (such as definition, goals, etc) to the developers. Also, we wanted to find out if the developers thought that the guidelines could potentially help them in creating gamified apps for self-management of chronic illnesses. Last, we wanted to know if the developers thought that implementing the Wheel of Sukr into an app would be feasible and if they could think of practical ways to gamify apps based on the guidelines. The selected developers are PhD students and postdoctoral researchers in computer science, Web technology, and software engineering at the University of Southampton. When a candidate participant was approached, they were asked if they had experience in developing apps to be included in the study, regardless of their level.

In this study, 5 focus groups were conducted. Each one consisted of 2 to 4 developers, resulting in 15 participants. After conducting the 5 focus group interviews, a point of saturation was reached where no new data were found. The main criterion for choosing the developers to be included in the sample is that they have experience in developing apps.

Each focus group session started with an overview of the research. Next, developers were presented with the guidelines to read. After reading each theme, the developers were asked to rate the guidelines for that theme based on 3 aspects: clarity, usefulness, and ease of implementation. In particular, they were asked to choose a score from 1 to 9 (1 being negative and 9 being positive). Then, they were asked to discuss the guidelines and raise any concerns about the content.

At the end of each focus group session, snapshots of an app for self-management of diabetes, which was arbitrarily chosen and does not contain gamification, were presented to the developers. They were asked to use the guidelines to suggest improvement to the app. The interviews were audiorecorded after getting the consent of participants, transcribed, and analyzed based on themes of the Wheel of Sukr. Finally, ethical approval was obtained from the ethics committee at the University of Southampton prior to conducting the interviews (reference number 20757).

## Results

### Expert Interviews

#### Overview

The expert interviews were aimed at confirming that the Wheel of Sukr guidelines as a whole are comprehensive, clear, and reflect the framework (the Wheel of Sukr).

Experts acknowledged the importance of introducing such guidelines. They provided a number of suggestions that were taken into account. Overall, experts thought that the guidelines provide good guidance for developers and have enough information to help them in this area. Moreover, they said that the way the guidelines are arranged flows nicely. The expert comments and feedback are divided based on the themes of the framework.

#### Fun

The discussion showed that there is a general consensus amongst the experts that this part of the guidelines is understandable, easy to use, and comprehensive. They also agreed that the points discussed in the guidelines are fundamental in making the self-management experience fun and rewarding. Moreover, the experts agreed that the guidelines are general enough to be used in creating different apps. As one expert stated, “It is very clear and it is not very specific that it can only be applied to a single case which is good.”

Nevertheless, one issue that needed clarification is the use of competition in the context of chronic illnesses self-management. The competition should not be associated with the self-managing tasks themselves or the results of the medical tests but rather with the number of times the user interacts with the system or the level of engagement the user has with the community, thereby gamifying the experience of self-managing. Consequently, a clarification remark was added to the competition element in the guidelines.

Some experts suggested considering the use of other core dynamics or other manifestations of the collection core dynamic. From a game design point of view, the badges and points are manifestations of the core game design collection. This manifestation is the most used in gamification in general. However, this does not mean that developers are limited to this form of core design. In light of this finding, the guidelines for the fun theme were improved. Other core design elements were mentioned in a way that is still true to the research and the framework of rewarding the user, not just creating a game-like experience. It is important to keep in mind the goal of the fun part of the guideline, which is to make the experience of self-managing chronic illnesses efficient while being enjoyable and positive.

#### Esteem

There was strong support for this part of the guideline and the way in which it is presented. This is evident when one expert said, “The way you described how they [the elements] need to be implemented in terms of the leaderboard and the progress bar is a very coherent way to represent how to encourage esteem both in the community (the external) and the internal in terms of how the person sees himself in that community.”

One point of clarification is that it is important to consider what the users might not want to share with others. For example, in self-managing diabetes some people might not be comfortable sharing their blood glucose levels. This issue was raised by one of the experts, and the guidelines were modified accordingly.

Another expert stressed the importance of creating the respect and admiration feelings for the user: “I believe this is extremely important because of the way that the user needs to know their progress and keep track.” The expert also mentioned the value of having reputation in the community: “It is also important to enable the user to be recognized by the community as a ‘super user’ or something like that.”

Moreover, it was pointed out that in the chronic illnesses communities the content of this theme is particularly useful. This is due to the fact that it allows those who do well in self-management to be an example to others and inspire them without pointing out to other users that you have to be like him or her. As the expert said, “Those who keep track of their self-management activities and do well, they will become an example and an encouragement to others in the community, and it will happen naturally.” Additionally, on using an app for self-management that has the elements of the esteem theme, the expert said, “In this environment the motivation could be even stronger than that of [other entertainment games] because it is related to users’ health.”

#### Growth

The impression about this part of the guidelines was very positive. Experts acknowledged that the guidelines are easy to understand, comprehensive, and reflect the Wheel of Sukr. This is exemplified in what one of the experts stated: “I agree with the elements that you got...and the way you want to link the system to the point and badges so that the person can see himself or herself growing in terms of changing their behavior and start having more control.”

Regarding the elements achieving goals and baby steps (tiny habits), one expert said, “We need a combination of difficult and easy tasks for the user, and the level of difficulty needs to increase in order to retain engagement. This is because if the user starts becoming an expert in what they do and they managed to change a tiny habit then surely you want to increase the difficulty.”

#### Motivation

The experts agreed on the comprehensiveness and clarity of the content of this guideline. One expert talked about linking intrinsic motivation to the app or system to help users understand that the reason they are feeling better is because of what they are doing in the app. This could be done through “...prompting people you can imagine having things like ‘Oh this week you managed to do better than you did last week.’”

Moreover, one of the experts discussed the age aspect and said, “I can see this working for both children and adults. The badges work for all age groups. Perhaps the colors would change and the theme but the elements work well for all the ages.”

Overall, the motivation guidelines were clear and comprehensive. This is exemplified in what one of the expert said: “You have considered two very important components of motivation. The one that comes from within and the one that I can develop from either what I see or that can hopefully feed my inner motivation.” The expert continued by saying that using both type of motivations would lead to increased engagement (“in something that is interesting and fun”), as well as allowing users to grow (“so that their inner self can be truly motivated and keep good health and the activities of self-management”). Thus, no changes were made to the motivation guidelines.

#### Sustainability

The common viewpoint among the experts was that the content of this guideline is comprehensive and clear. They expressed a strong view that this theme is a very important part of the guideline. Experts indicated the importance of reminding the user to use the app or to perform the self-management activities through using triggers, which could be in the form of a text or a sound.

The storyline and theme elements received the most attention. One expert expressed enthusiasm about the storyline element by saying, “It is very important—I really like this element in this theme. It is the way to actually make it [the experience of self-managing] meaningful. It creates the context where you can jump into that world and ‘find the magic.’” Another expert linked the story element back to intrinsic motivation: “In intrinsic motivation you seem like you are trying to say I want to point out to people that these things are being beneficial to their health, which you might communicate via story or you might communicate via some other means.” The expert continued saying that the storyline could be used as a motivator in this context.

Another expert suggested that we separate the definition of storyline and themes. Regarding the difference between the 2 elements, the expert said the theme is “the background that the user might connect to, to begin with,” while the storyline “is about controlling progress and the arc the player takes through their experience and on that note it might be important to think about what is the arc for the user for this system.” Furthermore, the expert discussed the way the developer will implement the storyline. They indicated that the developer must know the expected path the user will take to be able to manage their chronic illness in a good way. They must know the pace and the structure of the story that they are going to use. They also must consider the arc and structure of the story and how it will be connected to the game in order to create engagement. As the expert said, “The idea is that at the beginning you get the user or players attention and get them engaged in the experience, and then you relax that because you get impact with the user when you have acceleration. You have series of microclimatic before you have the big climax at the end and then relax.” Moreover, the standard design practice with regard to pacing and story structure should apply.

#### Self-Representation

The experts agreed that the content of this guideline is easy to understand and comprehensive. Expert agreed that it is important for the user to be able to change their avatar in a way that enables them to identify with the app. Giving users a way to express themselves would possibly increase their investment in the app.

The autonomy element is important in the context of self-managing chronic illnesses because giving the user control over their choices and activities could lead to patient empowerment. As one of the experts said, “How in control you are in a process, I can see how that is important to people managing conditions with these kinds of technology.”

#### Socializing

The general reaction to this theme was very positive as well. The experts thought that this part is straightforward, easy to understand, and comprehensive. As one of them said, “I agree with everything here, because I can see it on Twitter and social media you have groups for all chronic conditions and people get together, they support each other they understand they go through the same thing and they are there for each other.” The expert continued that peer support specifically is very important in the context of gamifying self-management of chronic illnesses. The expert said, “...we feel connected with someone we know understands because we are going through the same thing and it is different to hear it from someone who is speaking from a different place.”

#### Self-Management

The expert consensus on the guidelines for the self-management theme was that it is comprehensive and clear. As one expert said, “I agree with all the elements and especially the alert element, which I think is very important because it is required to help those who want to learn how to self-manage or to guide them on what to do.”

Regarding the alert element, experts agreed on its importance in the context of self-managing. As one expert said, “We tend to think about self-management oh you are independent you don’t need help, but this is not the case; it is just the person is prepared to know who to contact how to do what steps to follow to keep the condition under control. So alert is very important.”

One expert linked this theme to the esteem theme by saying, “You are essentially talking about communicating two types of information. One is about the status of the system and the game and it is covered by the esteem theme. The other one about the underlying status of their illness.”

### Focus Group

#### Overview

In this section, we present the findings of the focus group discussions with developers. As explained earlier, the developers were handed the set of guidelines and snapshots of a nongamified app for self-managing diabetes. They were asked to read the guidelines and discuss them theme by theme. After reading each theme, they were asked to rate it from 0 to 9 (0 the lowest rating and 9 the highest) in terms of clarity, usefulness, and ease of implementation. The average scores of each theme contained in the guideline are shown in Table 2. It should be noted that these scores reflect the opinions of developers on the guidelines. Clearly, the results show a very positive opinion toward the guidelines.

**Table 2:** Score table (ratings from 0-9).

	Clarity	Usefulness	Ease of implementation
Fun	7.5	7.7	7.1
Esteem	7.1	7.3	7.1
Growth	7.1	7.7	7.3
Motivation	6.1	7.7	5.5
Sustainability	7.8	8.1	7.5
Self-representation	7.5	7.8	7.9
Socializing	8.1	7.9	8.0
Self-management	8.3	8.2	8.4

#### Fun

One of developer said, “I have very little background of gamification but now I can read this and understand what these elements are and what I am supposed to do.” Another developer said, “Your guidelines adapt with what exist now [in the area of Web and app development] and it is very clear. ...our lives depend on collecting points and rewards.” It was evident that the fun theme elements are very clear as many of the developers managed to relate those elements to apps that they have been using, in particular health and fitness apps in which gamification aspects have been used.

The notion of sharing achievements between users, which was mentioned in the Application section of the fun theme, needed some clarification. In particular, the interviewer explained that the achievement element is not about sharing private medical results. Instead, it is about sharing the points collected or badges as a result of good self-management practices. Consequently, the guidelines were updated with this clarification.

Overall the developers were satisfied with the fun theme. One developer said, “From developers point of view I think these provide good guidelines; things to keep in mind while designing your app.” Another one said, “The guideline is general enough to help developers create different gamified apps.”

#### Esteem

The clarity of the theme is exemplified in what one developer stated: “I think it is clear and it goes well with the fun theme.” However, one point that needed clarification is the leaderboard. The interviewer explained that it is not calculated based on test results (eg, the blood glucose test results), but it is based on the activities of self-management—the tasks required (eg, the number of times the user entered their test results or the number of times the user achieved their goals). As indicated in the guidelines, the developer can add on this or change the leaderboard mechanism as long as they keep in mind the sensitivity of the data collected and not compare users based on their test entries (eg, their blood glucose levels).

#### Growth

One developer said, “This is for me quite useful and the description is clear,” which is in accordance with the general impression with the other developers as can be seen in Table 2. One point that needed clarification is that feedback does not mean feedback from other users; this type of feedback could be a part of the socializing theme as a matter of peer support. The feedback here is from the app itself. For example, when the user log their test results, a doctor character or another character can show up and reassure the user that they are doing a good job, or it could simply be a notification that appears containing a relevant message.

#### Motivation

Next, even though the ease of implementation for the motivation theme was low compared to the other themes, developers managed to come up with a number of examples on how to implement intrinsic and extrinsic motivations after some discussion. One of the examples given by developers was asking the user about their favorite animal and that would be their companion throughout the app. The only way to take care of their companion is by logging their data and performing the self-management tasks. Another example was to provide users with tips and information to maintain healthy lifestyle, which could enhance their motivation.

Overall, developers found the guidelines of the motivation theme useful. As one developer said, “The information is useful for the developer that there are 2 types of motivation that they can implement.”

#### Sustainability

Developers mentioned the challenge and difficulty of carrying on the elements of the sustainability theme and how the guidelines are helpful. Developers thought that if these elements were included in some of the apps they have used, they would have continued using them.

The elements trigger and nudge needed some clarification, as one of the developers could not distinguish between the two. Thus, the description of both elements in the guideline was edited to eliminate any future misconceptions. The trigger element is when a person is reminded to perform a behavior through visual or audio cues. On the other hand, the nudge is positive reinforcement and an indirect signal toward a nonforced act.

The satisfaction with this theme is summarized with the following statement: “The description of the storyline and theme is very helpful to me. And for the nudge it is useful because every time the user uses the app they get to enter their glucose which can help the users log everything daily and very intuitively. Also the reminders are useful for users, so if they forgot to use it they will remember. So I think this is very clear!”

#### Self-Representation

In this section, the ability element needed some clarification. Thus, it was clarified to show that when designing tasks or challenges, the developer should consider the varied abilities of users. For instance, some users might find it difficult to perform certain tasks. Therefore, simplifying the tasks is highly recommended. On using avatars, one developer said, “It creates a link between the user and app and lets the user engage with the app more.”

Overall, developers agreed that the self-representation part is clear (see Table 2). As one of the developers said, “It completely connects with what we have been discussing and I know how to implement everything here.”

#### Socializing

At this stage some developers started to see connections between the different themes of the whole guideline. As one developer stated, “This helps me understand the fun theme and esteem theme better, because it means people will share their achievements so they can engage more with the app.” Other developers supported the notion that the social aspect enables users to not only share their achievement with their peers but also with family and friends. This was summarized in the following statement: “It is useful to be connected with family, and they can see your progress and they will comment positively and then you will feel better.” Overall, the developers did not raise any issues regarding this theme and hence no modifications were made.

#### Self-Management

One of the developers said, “The points are quite clear, and I like the idea of adding the visualization because obviously they can clearly see the trends.” Another developer stated, “I think this is important. The logbook will help users check their progress, and the visualization would give users a straightforward impression on their progress.” The concepts contained in this theme were familiar to many of the developers, as some of them expressed that they have applied many of its elements in developing projects that they have worked on previously. No clarification was required for this theme, and hence the content of the guidelines has not been changed.

Finally, at the end of each focus group session developers were presented with the snapshots of a nongamified self-management app for diabetes, and they were asked if they could use the guidelines to suggest ways to implement gamification in this app. It was noted that developers were confident that the guidelines would enable them to transform a nongamified app into a gamified one. This includes those developers who had some misconceptions on some of the elements (before being clarified by the interviewer).

## Discussion

### Principal Findings

The use of gamification for health care purposes presents a tool that could enhance patient self-care [6,24,25]. Gamification could be thought of as a motivation tool and incorporates a number of behavioral change methods [5,26,27]. In the context of self-management of chronic illnesses, gamification could turn daily tasks of self-managing the illness into a rewarding and engaging activity [25]. However, as mentioned in the introduction, there is a shortage in developer guidelines. Current implementations of gamification in health care do not follow any specific guidelines [1]. Hence, this work fills the gap by providing a set of guidelines for developers.

This paper provides a set of guidelines for developers to gamify the self-management of chronic illnesses. The guidelines are based on the 8 themes of the Wheel of Sukr framework along with their elements [21,22]. The results from both studies, the expert interviews, and the developer focus group sessions show that the guidelines are clear, usable, easy to implement, and reflect the Wheel of Sukr. Specifically, the expert interviews ensure that the content of the guidelines reflect the framework and are comprehensive and sound. On the other hand, the focus group sessions with developers show the opinion of the end user of the guidelines (the developers) on the clarity, usefulness, and ease of implementation of the guidelines. After the expert interviews were conducted, the data were analyzed, and the guidelines were updated according to the findings. Subsequently, the guidelines were discussed in focus groups with developers, and the guidelines received a final update according to those findings.

The in-depth discussions with experts from academia in the fields of game and app design and user experience indicated that the guidelines cover adequate information. They also noted that the guidelines would be useful for developers of self-management apps. Moreover, the experts discussed the importance of the elements in the guidelines for users (specifically, the community aspect and how it can provide peer support and the flow and ability element where the different abilities of users are taken into consideration while designing the tasks). Furthermore, the focus group sessions with developers showed that the guidelines could be useful in creating gamified self-management apps for chronic illnesses. This was evident when developers were shown a self-management app that does not include gamification elements and they managed to use the guidelines to suggest specific ways to implement gamification in the app.

The methods chosen in this study are expert interviews and focus group sessions with developers. Interviews were chosen to enable in-depth discussion and assessment of the guidelines [28]. The interviewees came from different but relevant backgrounds. This puts them in a position to give vital feedback on the guidelines based on their expertise. Likewise, the input from developers was necessary to ensure that the target group of the guidelines (ie, developers) can comprehend the content of the guidelines and finds them useful and easy to work with. To accommodate the different levels and backgrounds of these developers, a focus group method was most suitable [29]. Indeed, the developers managed to discuss the guidelines among themselves and answer each other’s concerns and questions.

There was an agreement from participants in both studies that such guidelines are needed in this area. This is also supported by literature findings, where some researchers argued that current implementations of gamification in health care do not adhere to standard guidelines [3]. Additionally, it was suggested that gamified apps do not reflect the theoretical frameworks and approaches found in literature [1]. In their paper, Seaborn and Fels [1] argue that theoretical work is not studied empirically and the apps and systems that applied some of the theories did not test their validity empirically. However, given that gamification is still considered at an early stage in terms of being applied to the self-management of chronic illnesses, there must be a starting point, and a theoretical framework along with comprehensive guidelines is needed.

In order to find the strengths and weaknesses of the themes and their elements, an empirical study incorporating them within the context of self-managing chronic illnesses must be undertaken. This will help to determine the best practices in gamifying self-management of chronic illnesses (eg, which type of badges and triggers are most effective). This is a subject of a future study to be conducted by the authors.

### Conclusion

This paper presented guidelines for the development of gamified self-management apps and system for chronic illnesses. The Wheel of Sukr framework was translated into a set of guidelines for developers. The guidelines are divided into 5 parts: theme and their corresponding elements (from the Wheel of Sukr), definition, goal, description, and application. The content of the guidelines was discussed in depth with experts from academia using semistructured interviews. The experts had experience in the areas of game development, user experience, and gamification. The findings from the expert interviews suggest that the guideline content is comprehensive and reflects the Wheel of Sukr. Moreover, the experts gave suggestions to enhance the guidelines and those were taken into consideration to update the guidelines. After that, the updated version of the guidelines was discussed with developers in focus group interview sessions to ensure the clarity, usefulness, and ease of implementation. The findings of the focus group interviews show that there is an overwhelming agreement between developers that the guidelines are useful, easy to implement, clear, and can be applied to create self-management gamification apps.

On light of our findings, we believe that the Wheel of Sukr guidelines are ready to be tested practically in the creation of apps for the target patients. In fact, in a future study we aim to design such an app and test it on patients using a longitudinal method.

## Appendix

Multimedia Appendix 1 The Wheel of Sukr Guidelines.
