# Supplementary material for: Guidelines for the Gamification of Self-Management of Chronic Illnesses: Multimethod Study
Source: JMIR Serious Games. 2017 May 12;5(2):e12. doi: 10.2196/games.7472 (PMC5446669; doi:10.2196/games.7472)
Supplement: Multimedia Appendix 1 [file games_v5i2e12_app1.pdf]

Guidelines for Gamifying Self-management of Chronic illnesses

**Fun Theme (**Badges, points, challenges and competition**)**	
**Definition**	The Fun theme has the basic building blocks of any gamification system. It represents the rewarding features of gamification. The Fun theme enables the creation of a game like experience.
**Goal**	Create a rewarding and engaging experience.
**Description**	• The Badges element indicates the special designs that can be awarded to users as a sign of membership and achievement. It can be awarded to users once they start using the application, when accumulating a number of points, and when completing a level.<br>• The Points element indicates the points that are awarded to users when entering test results, completing the minimum required tests per day, completing a week of entering test results, completing a challenge, and when communicating with other users.<br>• The Challenges element indicates the motivators created in the gamified system to get users to engage in difficult but obtainable tasks. These could be completing a week of entering test results, or maintaining a certain level of glucose.<br>• The Competition element indicates the competitive environment that can be created using the elements in this theme and other themes (such as levels and leaderboards) to increase users engagement. The competitive environment should be exclusive to the activities completed (i.e. the number of times the user logged in, the user's involvement in the community, completing tasks… etc.) and not regarding their test results. Users can compete with each other in the community and with themselves.
**Application**	Design a system of rewards (points and badges) based on the community you are targeting and on the unique goals of the app/system.<br>Create challenges for users to increase their engagement and help them meet their goals.<br>Give users the option to view each other's achievements.<br>Design the competitions with the user in mind (i.e. do not create insensitive competitions regarding their test results). Make the experience positive and rewarding.<br>Remember that collecting badges and points is a manifestation of the "collection" Core Dynamics in games, and you could use different manifestation of collection.<br>➢ This theme is closely related to the Esteem and Motivation themes.

**Esteem Theme (**leaderboards, levels, progress bars, and reputation**)**	
**Definition**	The Esteem theme encompasses the essential elements to create respect and admiration feelings for the user. It communicates the state of the system to users.
**Goal**	Enable users to respect and admire their own achievements and the

Alaa Almarshedi © Copyright 2016
aaa3g12@soton.ac.uk

	achievements of others.
**Description**	• The Leaderboard element indicates the leaderboards that are used in video games. Similar to video games, in gamification leaderboards are social features used to increase engagement, motivation, and the level of competition between players. This is done by ranking them in a number of ways such as according to the number of points or badges collected or the levels achieved… etc. There are different types of leaderboards. Such as global leaderboards, which ranks the top users of the application, and friends or social leaderboards that rank the user's friends or who are in their social network (Twitter, Facebook... etc.).<br>• The Progress Bar element is a graphical element in the user interface that shows the progression of the user and how close he/she is to completing a set of tasks. It could be in a percentage, visual form or textual representation.<br>• The Levels element indicates the part of the application that users need to complete to meet specific goal to advance to the next level. The difficulty of every level increase as the user complete new levels.<br>• The Reputation element indicates the user's status, rank and expertise in the community. It is the way users showcase their points and badges. Reputation mechanics must be designed and updated based on the community.
**Application**	Design leaderboards and choose the type or types of leaderboards that fit the community and the app/system goal.<br>Create a progress bar that measures the users activity based on the goal of the app. For example, measure the user's involvement in the community or the user's progress in logging their test results.<br>Design levels based on the goal of the app, considering the Storyline and Theme elements (from the Sustainability theme), and the Rewards and Competitions elements (from the Fun theme).<br>Design Feedback, Leaderboards, Progress Bar, Levels and the Community (from the Socializing theme) coherently to utilize the reputation mechanics where the user can showcase their status in the community.<br>Consider the sensitivity of the issue with regards to social gaming, and the level of confidentiality involved.

**Growth theme (**feedback, achieving goals, and baby steps (tiny habits)**)**	
**Definition**	The growth theme represents the elements that aid the users in growing and developing in their self-management of their chronic illness.
**Goal**	Empower patients to take good care of their health by learning about their illness and learning to manage it.
**Description**	• The feedback element indicates the comments users receive regarding their test results entries and their overall performance in the application (their self-management habits).<br>• The Achieving Goals element indicates outlining clear goals and rules in the system. Goals could also be set by the user or with the help of

	caregivers. It could be regarding self-monitoring blood glucose, such as the frequency of blood testing and whether the patient keeps a log of the tests or not.<br>• The Baby Steps/Tiny Habits element indicates breaking down activities into a sequence of smaller tasks that are easy to incorporate in the users' daily routine. This is believed to result in adoption of the new "tiny habits" to reach a desired behaviour. This helps in changing behaviour in self-management. For instance, a diabetic patient should not be expected to change in a matter of days.
**Application**	Design a feedback system based on the goal of the app/system.<br>Link the feedback system to the points and badges.<br>Set goals to users to encourage them to start.<br>Give users the option to set their own goals.<br>Remember that breaking the goals, challenges into smaller steps would be easer for users to adopt.<br>Do not make tasks and gaining points and badges too difficult to the user to avoid decreasing their engagement and interest in the app/system. Increase the difficulty as their experience increase.<br>➢ This theme is closely related to the self-management theme.

**Motivation theme (**intrinsic motivations, extrinsic motivations**)**	
**Definition**	The motivation theme includes motivators that are innate to the user and extrinsic motivators. Both types of motivators are important in the design of gamified apps/systems.
**Goal**	Utilize extrinsic motivators to increase intrinsic motivation.
**Description**	• The intrinsic motivation element indicates the innate desire to perform an activity for the love and joy that it brings. Intrinsic motivators could include health, social satisfaction, status, mastery, purpose, acceptance, and curiosity.<br>• The extrinsic motivation element indicates the performance of an activity solely for the external rewards, which could include points, rewards, and gaining status. It has four types:<br>o External regulation: results from outward rewards or punishments.<br>o Introjection: results from self-esteem issues such as doing something to avoid guilt or anxiety, or to boost their ego.<br>o Identification: occurs when a person self-identifies with the importance of a behaviour, and has accepted its regulation as their own.<br>o Integration: external motivation that has been internally integrated by the user. This type of motivation shares qualities with intrinsic motivation, such as autonomy and being unconflicted.
**Application**	Know your target audience and what motivates them.<br>Remember that the self-management tasks that patients should do may not

Alaa Almarshedi © Copyright 2016
aaa3g12@soton.ac.uk

	feel inherently interesting or enjoyable to them. This could be due to the lack of perceived autonomy and competence, which are essential for intrinsic motivation.<br>Utilize the types of extrinsic motivations that could promote more active and volitional (as opposed to passive and controlling) forms of extrinsic motivation are essential.

**Sustainability theme (**trigger, flow, storyline and theme, and nudge**)**	
**Definition**	The Sustainability theme includes the element that ensures the longevity and continuous user engagement and interest.
**Goal**	Ensure the longevity of the gamified effect and encourage consistent and better self-management habits.
**Description**	• The Trigger element indicates the visual or audio messages or cues that could trigger behavior. In the context of self-management of diabetes, a trigger could be a message to the user to remind him/her to enter test results, or communicate with others... etc.<br>• The Flow element represents the mental state of absorption and engagement in an activity or a game. In the flow state, the user is intrinsically motivated and completely immersed in what they are doing. The gamified activities in the self-management application should be designed according to users' skill level. Thus, gradually increasing the difficulty as the users' skills increase.<br>• The Storyline and Theme elements indicate the importance of creating a link between the user and the application. The theme is the background that the user might connect to at the beginning. Whereas the storyline is about controlling the progress and the arc the user takes through the experience. These elements also give more meaning to the gamification features. Creating a journey that makes the user part of it and increases their engagement.<br>• The Nudge element is based on one of the theories known to drive behaviour The Nudge theory. The nudge is the use of positive reinforcement and indirect signals to lead to a non-forced action, by creating the simplest path to certain behaviour.
**Application**	Create a bond with the user through introducing a theme and constructing a storyline to the app/system and create a journey.<br>Design reminders to the user to log their daily test results by using triggers such as sound cues or messages.<br>Nudge the user into the desired behaviours you want them to undertake. For example, showing the logbook once they sign into the app to encourage them to fill it first.

**Self-representation (**avatars, autonomy, ability, and purpose**)**	
**Definition**	The self-representation theme signifies the importance of creating a link between the user and the app.
**Goal**	Enhance the gamification experience and efficiency of the self-

	management.
**Description**	• The avatar element means providing the user with means to express themselves in terms of allowing them to upload profile images, offers predesigned images for them to choose from, or allow them to personalise their profiles.<br>• The autonomy element is when the users have full control over their choices and activities.<br>• The Ability element suggests that developers should consider the varied abilities of users, and that they should simplify the tasks required.<br>• The Purpose element indicates that the goal and purpose of self-management is clear to the user.
**Application**	Give the user options to express them selves in the system to help them create a bond with the system.<br>Remember that to keep and enhance the user intrinsic motivation, they must experience their behavior to be autonomous.<br>Design the challenges, tasks and other gamification features in an obtainable way.<br>Clarify the importance of the tasks and activities in the app/system to users.

**Socializing theme (**community, social media, peer-support, and sharing**)**	
**Definition**	The socializing theme covers the social and emotional aspect of self-managing diabetes.
**Goal**	Provide users with a community that creates a base for the gamification features and offer the emotional and social support for diabetics.
**Description**	• The Community element means creating a network that is held together by the shared experience of having chronic illness and the common goal of living a healthy life.<br>• The Social Media element means providing the user with the choice to link his/her self-management profile or activities with their other social media accounts such as Facebook or Twitter.<br>• The Peer-Support element indicates the support that is provided through the community (other users in the app), the social media (if the app is linked to social media such as Twitter and Facebook), and the gamified features such as feedback and rewards.<br>• The Sharing elements means providing the user with the option to share their test results, progress… etc., with others in the community.
**Application**	Create a community where users can share their experiences, and learn coping strategies from each other.<br>Allow users to link their profiles to their social media profiles. In which they can share their achievements and rewards with their family and friends who are not in the community.<br>Link the Fun Theme, Esteem Theme, and Sustainability Theme elements with the community and social media features to enhance the value of

Alaa Almarshedi © Copyright 2016
aaa3g12@soton.ac.uk

	rewards, challenges, and competition.

**Self-management (**logbook, visualization of data, and trend alerts**)**	
**Definition**	The self-management theme covers the essential elements of a diabetes self-management app/system.
**Goal**	Assist users in self-managing their illness in an efficient way.
**Description**	• The Logbook is a space that allows the user to keep logs on their test results, date/time, food intake, exercise, how they are feeling, and other relevant information.<br>• The Visualization of Data element indicates turning the data in the user's logbook into visual representations. This way the user can benefit from the accumulated information. For example, a user can see how well they did in the past week or month using a table or a specific graph. Also, this would enable them to spot patterns easier.<br>• The Trend Alert element indicates the use of the logged data to alert the user of any sudden changes or worrying results such as extreme lows/highs in their blood glucose… etc.
**Application**	Create a logbook where users can enter all the data they need to save from their daily medical test results. For example: blood glucose level, insulin injection, psychological state, food intake, and activities.<br>Create graphs and charts from the data saved by the user. Present these graphs in weekly, monthly, and yearly options.<br>Alert the user of any trends such as very low/high blood glucose or if the user did not enter their test results of the day.<br>Consider the privacy of the user's data.
